# Safety and tolerance of oral ketamine: A systematic review

**DOI:** 10.1111/pcn.70091

**Published:** 2026-05-30

**Authors:** Nathalia Novaretti, Rebeca Mendes P. Pessoa, Jaime Eduardo Cecílio Hallak, Rafael Guimarães dos Santos, Marcos Hortes N. Chagas

**Affiliations:** ^1^ Department of Neurosciences and Behavior School of Medicine of Ribeirão Preto, São Paulo University Ribeirão Preto São Paulo Brazil; ^2^ National Institute of Translational Science and Technology in Medicine (INCT‐TM), CNPq Porto Alegre Brazil

**Keywords:** adverse effects, ketamine, Oral administration, safety, tolerability

## Abstract

Oral ketamine has gained increasing interest beyond anesthesia, particularly for treatment‐resistant depression, chronic pain, and pediatric procedural sedation. Despite expanding off‐label use, there is no standardized pharmaceutical formulation, and long‐term safety remains uncertain. A synthesis of evidence on safety and tolerability of the oral route is therefore needed to inform clinical practice and regulatory development. This systematic review evaluated the safety and tolerability profile of oral ketamine in humans and characterized dosing strategies across randomized controlled trials. A systematic search was conducted in PubMed, Embase, Scopus, and Web of Science from inception to November 2025 (PROSPERO CRD420251065295). Randomized placebo‐controlled trials evaluating oral (es)ketamine in any clinical population were included. Primary outcomes were safety and tolerability. Risk of bias was assessed using the RoB2 tool, and certainty of evidence was evaluated using the Grading of Recommendations Assessment, Development and Evaluation (GRADE) framework. Eighteen trials were included, comprising adults, children, and healthy volunteers. In depression studies (*n* = 5; 427 participants), adverse effects were predominantly mild and transient. Pediatric premedication trials (*n* = 239) reported transient neurological effects without clinically significant safety concerns. Pain and experimental studies (*n* = 372) showed mostly mild adverse events, with dissociative symptoms at higher doses. Comparative analyses indicated higher rates of dizziness, sedation, and dissociative symptoms with ketamine, while serious adverse events were rare. Overall, oral ketamine demonstrates a favorable short‐term safety profile. However, the certainty of evidence is low, and long‐term safety remains uncertain.

Ketamine was first synthesized in the early 1960s and introduced into clinical practice as a short‐acting dissociative anesthetic administered intravenously or intramuscularly.^1^ Due to its unique pharmacological profile – providing analgesia, amnesia, and anesthesia while preserving airway reflexes and spontaneous respiration – it rapidly became a valuable agent in surgical and emergency settings.^2^, ^3^ Traditionally, ketamine has been used parenterally; however, its administration *via* the oral route has attracted growing interest because of its ease of use, lower cost, and potential suitability for outpatient and palliative care environments.^4^

Oral ketamine has been explored not only as a sedative but also as an adjunct for procedural sedation and pain control in both acute and chronic settings.^5^, ^6^ The bioavailability of oral ketamine is lower compared to intravenous administration, but its active metabolite, norketamine, may contribute significantly to its clinical effects.^4^ Importantly, there is currently no commercially available oral formulation of ketamine, and its oral use remains off‐label, relying on compounded preparations or repurposed injectable solutions.^7^ Despite these limitations, the oral route has been increasingly investigated due to its potential practicality and patient acceptability, especially for chronic treatment.

In recent years, the therapeutic use of ketamine has expanded beyond anesthesia to include psychiatric and pain disorders. Oral formulations have been studied as potential treatments for chronic pain syndromes and treatment‐resistant depression,^8^, ^9^ conditions in which conventional therapies may be ineffective or poorly tolerated.^10^, ^11^ Nevertheless, uncertainties persist regarding the safety profile and tolerability of oral ketamine, particularly concerning neuropsychiatric, cardiovascular, and hepatic adverse events, as well as its potential for misuse and dependence.

Despite the logistical and cost advantages of the oral route, its use remains largely off‐label and depends on nonstandardized compounded preparations.^7^ Furthermore, although intravenous ketamine and intranasal esketamine have received regulatory approval for specific indications, evidence regarding the safety and tolerability of oral ketamine remains fragmented and heterogeneous. Previous systematic reviews, including those by Rosenblat *et al*. and Meshkat *et al*.,^8^, ^9^ primarily focused on the antidepressant efficacy of ketamine, often including open‐label studies, observational designs, or mixed routes of administration. For example, earlier reviews evaluating oral ketamine in depression synthesized preliminary clinical evidence but frequently incorporated non‐randomized studies and primarily assessed therapeutic efficacy rather than safety outcomes. Consequently, the safety profile of oral ketamine across different clinical contexts remains insufficiently characterized.

In contrast, the present systematic review specifically evaluates the safety and tolerability of oral ketamine in humans, across multiple clinical settings, and restricts inclusion to randomized controlled trials (RCTs) in order to provide a more rigorous assessment of treatment‐emergent adverse events. By focusing on randomized evidence and including indications beyond depression, this review aims to clarify the current safety evidence base and explain why fewer studies were included compared with prior reviews despite a more recent search period.

## Methods

### Literature search and study selection

Two independent reviewers (NN and RP) searched the literature for studies that evaluated ketamine treatment using oral administration. We searched PubMed, Embase, Scopus, and Web of Science from inception to 11 November 2025. Search terms included a combination of Medical Subject Headings and text words indicative of: (i) (es)ketamine; (ii) oral use; and (iii) adverse effects. The full electronic search strategy for all databases is provided in Appendix I. An additional search was performed in the reference list of identified articles. There were no language restrictions. Our systematic review reported results using the Preferred Reporting Items for Systematic Reviews and Meta‐Analyses (PRISMA) guidelines^12^ and methods were preregistered (PROSPERO, CRD420251065295).

### Study outcomes

The primary outcomes were to evaluate the safety profile of orally administered ketamine and to assess its tolerability and associated adverse effects. Secondary outcomes included characterizing the dosing regimens used for oral administration.

### Eligibility criteria

Eligibility criteria were established using the PICO framework (Population, Intervention, Comparator, and Outcome). Specifically: Population (P) included human participants with any clinical condition (e.g., depression, chronic pain) or healthy volunteers; Intervention (I) was oral ketamine (racemic or esketamine, immediate or extended release); Comparator (C) included placebo; and Outcomes (O) of interest were safety (serious adverse events, changes in vital signs, hepatotoxicity, urological symptoms) and tolerability (all‐cause discontinuation, subjective adverse events, dissociative symptoms). Only randomized, RCTs. The decision to include only RCTs was primarily intended to ensure a high level of internal validity and allow for a direct comparison of treatment‐emergent adverse events against a control group across highly heterogeneous clinical populations and dosing regimens. While this approach prioritizes the mitigation of confounding factors, we acknowledge it may limit the detection of rare or delayed adverse events typically captured in larger observational cohorts. The exclusion criteria were studies that did not report data on safety, tolerability, or adverse effects; studies using routes of ketamine administration other than oral; and conference abstracts or clinical trials without published data.

### Study selection

Authors NN and RP reviewed all articles that met inclusion criteria. The screening and selection of studies were performed using Rayyan, an online tool designed to facilitate systematic review processes.^13^ Data were extracted using a standard data extraction form for: sample size, intervention features (i.e., method of assessment, details of controls), mean age, study population, and outcomes related to tolerance and safety.

### Assessment of bias

Study quality (i.e., risk of bias) was assessed using the revised Cochrane risk‐of‐bias tool for randomized trials.^14^ Bias was assessed in accordance with the six domains evaluating risk of bias: bias arising from the randomization process, bias due to deviations from intended interventions, bias due to missing outcome data, bias in measurement of the outcome, bias in the selection of the reported result, and bias arising from conflicts of interest. Disagreements were resolved by consensus. Complete results from the Cochrane risk‐of‐bias guidelines are presented in Table 1. Furthermore, this systematic review strictly adhered to the Preferred Reporting Items for Systematic Reviews and Meta‐Analyses (PRISMA) 2020 Guidelines.

**Table 1:** Cochrane risk of bias assessment results

Source	Domain 1: Risk of bias from the randomization process	Domain 2: Risk of bias due to deviations from the intended interventions	Domain 3: Risk of bias due to missing outcome data	Domain 4: Risk of bias in measurement of the outcome	Domain 5: Risk of bias in the selection of the reported result	Overall assessment of bias
Qureshi *et al*., 2025^19^	**↓**	**↓**	**↓**	**↓**	**↓**	**↓**
Dinsmore *et al*., 2025^27^	**↓**	**↔**	**↔**	**↔**	**↓**	**↔**
Glue *et al*., 2024^16^	**↔**	**↓**	**↔**	**↓**	**↓**	**↔**
Smith‐Apeldoorn *et al*., 2024^11^	**↓**	**↓**	**↔**	**↓**	**↓**	**↔**
Colla *et al*., 2024^15^	**↓**	**↔**	**↔**	**↓**	**↔**	**↔**
Katiyar *et al*., 2022^30^	**↓**	**↓**	**↓**	**↓**	**↔**	**↔**
Glue *et al*., 2020^31^	**↓**	**↓**	**↓**	**↓**	**↓**	**↓**
Rayala *et al*., 2019^28^	**↓**	**↔**	**↓**	**↔**	**↔**	**↔**
Domany *et al*., 2019^21^	**↓**	**↔**	**↔**	**↓**	**↓**	**↔**
Arabzadeh *et al*., 2018^20^	**↓**	**↓**	**↔**	**↔**	**↔**	**↔**
Barkan *et al*., 2014^29^	**↓**	**↓**	**↓**	**↓**	**↓**	**↓**
Kararmaz *et al*., 2004^24^	**↓**	**↓**	**↔**	**↓**	**↓**	**↔**
Turhanoğlu *et al*., 2003^22^	**↓**	**↓**	**↓**	**↔**	**↓**	**↔**
Mikkelsen *et al*., 2000^17^	**↓**	**↓**	**↓**	**↔**	**↓**	**↔**
Rabben *et al*., 1999^18^	**↓**	**↔**	**↔**	**↔**	**↔**	**↔**
Şekerci *et al*., 1996^25^	**↓**	**↓**	**↔**	**↔**	**↓**	**↔**
Qureshi *et al*., 1995^26^	**↓**	**↓**	**↓**	**↔**	**↓**	**↔**
Gutstein *et al*., 1992^23^	**↓**	**↓**	**↓**	**↔**	**↓**	**↔**

**↓** Low risk of bias; **↔** Some concerns; and **↑** High risk of bias. No study was judged to be at high risk of bias across overall RoB2 domains.

### Data synthesis

Due to substantial heterogeneity in study populations, clinical indications, ketamine formulations, dosing regimens, and outcome reporting, a quantitative meta‐analysis was not considered appropriate. Included studies varied widely across clinical contexts, including treatment‐resistant depression, postoperative pain, pediatric pre‐anesthetic medication, and experimental studies in healthy volunteers. In addition, adverse events were reported using heterogeneous methods, ranging from structured scales to spontaneous reporting, preventing reliable pooling of event rates.

Therefore, results were synthesized using a structured narrative approach, consistent with methodological guidance for systematic reviews where statistical pooling is not feasible. When comparable adverse‐event counts were available, descriptive pooled proportions and exploratory risk ratios were calculated when appropriate to provide comparative context between oral ketamine/esketamine and placebo. Because of substantial clinical and methodological heterogeneity, these estimates were not treated as formal meta‐analytic pooled effects, and *I* ^2^ statistics were not calculated.

### Certainty of evidence assessment

The certainty of evidence for key safety outcomes was evaluated using the Grading of Recommendations Assessment, Development and Evaluation (GRADE) framework (GRADE Working Group). Evidence from randomized controlled trials was initially rated as high certainty and subsequently downgraded based on risk of bias, inconsistency, indirectness, imprecision, and publication bias. A Summary of Findings table was generated to summarize the certainty of evidence across the main adverse‐event outcomes (Table 2).

**Table 2:** GRADE summary of findings: Safety and tolerability of oral ketamine compared with placebo

Outcomes	No. of participants (studies)	Certainty of the evidence (GRADE)	Explanations
Any Adverse Event	1015 participants (18 RCTs)	⊕ ⊕ ◯◯ LOW^†^, ^‡^	Downgraded due to risk of bias (unstructured adverse event reporting in several trials) and imprecision (small sample sizes and low event frequency).
Neurological and neuropsychiatric adverse events (e.g., dizziness, dissociation, sedation)	Up to 1015 participants (18 RCTs)	⊕◯◯◯ VERY LOW^†^, ^‡^, ^§^	Downgraded due to risk of bias (lack of standardized assessment tools), inconsistency (variability across studies related to dosing regimens and populations), and imprecision.
Gastrointestinal AEs (e.g., nausea, vomiting, dry mouth)	Up to 1015 participants (18 RCTs)	⊕ ⊕ ◯◯ LOW^†^, ^‡^	Downgraded due to risk of bias (detection bias due to spontaneous reporting) and imprecision.
Cardiovascular safety (blood pressure and heart rate changes)	Up to 500 participants (10 RCTs)	⊕ ⊕ ⊕◯ MODERATE^‡^, ^††^	Downgraded due to imprecision. Not downgraded for risk of bias as outcomes were assessed using objective physiological measurements.
Discontinuation due to adverse events	427 participants (5 RCTs)^‡‡^	⊕ ⊕ ◯◯ LOW^‡^, ^¶^	Downgraded due to imprecision related to the very low number of discontinuation events.
Serious Adverse Events	1015 participants (18 RCTs)	⊕ ⊕ ◯◯ LOW^‡^, ^¶^	Downgraded due to imprecision (rare events in relatively small samples) and indirectness related to short exposure duration.

^†^ Risk of bias: downgraded due to the use of non‐validated instruments or spontaneous adverse event reporting in several trials.

^‡^ Imprecision: downgraded due to small sample sizes and low event rates, limiting the ability to detect rare adverse effects.

^§^ Inconsistency: downgraded due to variability across studies related to differences in dosing regimens, formulations, and clinical populations.

^¶^ Indirectness: downgraded due to short follow‐up periods, limiting inference about long‐term safety outcomes.

^††^ Objective measurement: cardiovascular outcomes were assessed using physiological parameters such as blood pressure and heart rate monitoring.

^‡‡^ Discontinuation data: Based on trials in major depressive disorder (*n* = 427) where treatment‐emergent adverse events leading to withdrawal were systematically reported.

## Results

### Search results and study characteristics

**Fig. 1:**
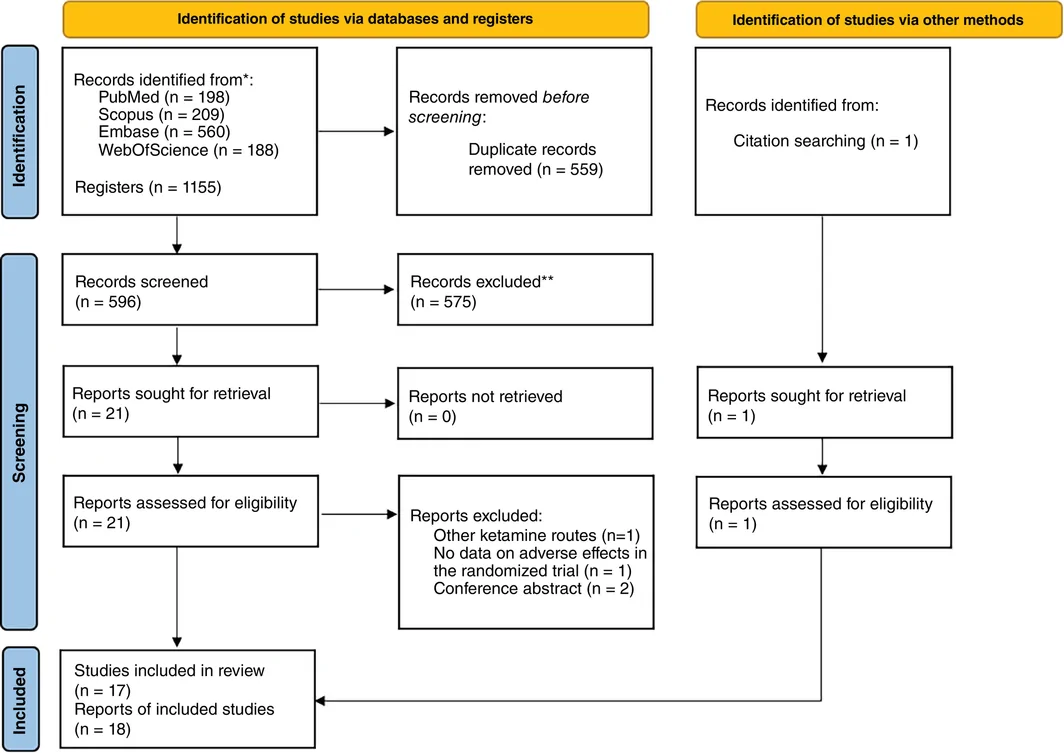
PRISMA flow diagram of study selection. Flow diagram illustrating the process of study identification, screening, eligibility assessment, and inclusion of randomized placebo‐controlled trials evaluating oral ketamine. A total of 1155 records were identified through database searching, with 21 full‐text articles assessed for eligibility and 18 studies included in the final analysis.

A total of 1155 records were identified through database searching. After removal of duplicates, 596 records remained. Following title and abstract screening, 575 records were excluded. Twenty‐one full‐text articles were assessed for eligibility, of which 17 met the inclusion criteria. In addition, one article was identified through citation searching of the included studies. In total, 18 studies were included in the review. A complete summary of search results is presented in accordance with the PRISMA guidelines (Fig. 1).

Three trials were multicenter,^11^, ^15^, ^16^ and the remaining were conducted in single centers. Two studies used a crossover design,^17^, ^18^ and one used a partial crossover design.^19^ Five trials investigated oral ketamine in major depressive disorder,^11^, ^15^, ^16^, ^20^, ^21^ four evaluated its use as pediatric pre‐anesthetic premedication,^22^, ^23^, ^24^, ^25^ six examined analgesic applications,^18^, ^26^, ^27^, ^28^, ^29^, ^30^ and three were conducted in healthy volunteers.^17^, ^19^, ^31^ Only one study evaluated oral esketamine^11^; all others used racemic ketamine. The oral formulations were heterogeneous: four trials used capsules, four used extended‐release tablets, and the remainder used liquid solutions. Dosing strategies ranged from single‐dose protocols designed for procedural sedation or premedication to multi‐week regimens for depression and chronic pain. Nine studies used a single administration, while the rest used repeated dosing schedules. Detailed characteristics of the included studies are presented in Table 3.

**Table 3:** Characteristics of ketamine administration of included randomized clinical trials

Source	Formulation	Investigated condition(s)	Method of administration	Total daily dose	Dose frequency
Qureshi *et al*., 2025^19^	Racemic	Healthy subjects	Capsule	40–120 mg or 160–240 mg	Once weekly for 3 weeks
Dinsmore *et al*., 2025^27^	Racemic	Pain	Liquid	90 mg	Every 8 h for 3 days (9 total doses)
Glue *et al*., 2024^16^	Racemic	Major depression	Extended‐release formulations	30, 60, 120 or 180 mg	Twice weekly for 12 weeks
Smith‐Apeldoorn *et al*., 2024^11^	Esketamine	Major depression	Capsule	30–90 mg	Three times daily for 6 weeks
Colla *et al*., 2024^15^	Racemic	Major depression	Extended‐release formulations	160 or 240 mg	Twice daily for 14 days
Katiyar *et al*., 2022^30^	Racemic	Pain	Liquid	3 mg/kg	Single dose
Glue *et al*., 2020^31^	Racemic	Healthy subjects	Extended‐release formulations	60, 120 or 240 mg	Single dose followed by every‐12‐h dosing for 5 doses
Rayala *et al*., 2019^28^	Racemic	Pain	Liquid	0.8 mg/kg	Single dose
Domany *et al*., 2019^21^	Racemic	Major depression	Liquid	1 mg/kg	Three times per week for 21 days
Arabzadeh *et al*., 2018^20^	Racemic	Major depression	Capsule	50 mg	25 mg every 12 h for 6 weeks
Barkan *et al*., 2014^29^	Racemic	Pain and sedation	Liquid	5 mg/kg	Single dose
Kararmaz *et al*., 2004^24^	Racemic	Pre‐anesthetic medication	Liquid	6 mg/kg	Single dose
Turhanoğlu *et al*., 2003^22^	Racemic	Pre‐anesthetic medication	Liquid	4, 6 or 8 mg/kg	Single dose
Mikkelsen *et al*., 2000^17^	Racemic	Pain	Liquid	0.5 or 1 mg/kg	Single dose
Rabben *et al*., 1999^18^	Racemic	Pain	Capsule	4 mg/kg	Once daily for 3 days
Şekerci *et al*., 1996^25^	Racemic	Pre‐anesthetic medication	Liquid	3 or 6 mg/kg	Single dose
Qureshi *et al*., 1995^26^	Racemic	Pre‐anesthetic medication	Liquid	10 mg/kg	Single dose
Gutstein *et al*., 1992^23^	Racemic	Pre‐anesthetic medication	Liquid	3 or 6 mg/kg	Single dose

### Safety and tolerability in major depression

Across the five depression trials, 427 adults with major depressive disorder received oral ketamine. The mean age was 43.2 years, and daily doses ranged between 30 and 240 mg. The studies differed notably in formulations: two used extended‐release preparations,^15^, ^16^ one used standard immediate‐release capsules,^20^ one used a liquid formulation,^21^ and one study used oral esketamine capsules.^11^ Adverse‐event assessment varied, although the more recent trials^11^, ^15^, ^16^ consistently used validated scales.

Adverse events were generally mild across studies. The symptoms most frequently reported included dizziness, postural dizziness, nausea, dry mouth, and blurred vision, in addition to transient elevations in liver enzymes. Importantly, three trials found no statistically significant difference in overall adverse‐event incidence between ketamine and placebo.^15^, ^20^, ^21^ Only Smith‐Apeldoorn *et al*.^11^ observed a higher rate of dizziness and orthostatic symptoms in the ketamine group. Five participants discontinued treatment due to adverse events: one due to mild‐to‐moderate somnolence,^21^ one due to worsening of depressive symptoms, one due to anxiety, one due to generalized rash, and one due to a constellation of symptoms (anxiety, headache, dizziness, dry mouth, and nausea).^11^ Three serious adverse events were documented – one suicide, one lens dislocation, and one case of diverticulitis – none attributed to the study drug.^11^, ^15^ The extended‐release trial by Glue *et al*. contributed additional safety information, reporting severe headache, transient depressive worsening, non‐cardiac chest pain, nausea, intervertebral disc protrusion, and urinary tract stones, although serious events occurred in both active and placebo groups and were deemed unrelated to treatment^16^ (Table 3).

### Safety and tolerability of pre‐anesthetic premedication

Four trials assessing oral ketamine as pre‐anesthetic premedication enrolled 239 children.^22^, ^23^, ^24^, ^25^ Of these, 158 participants received oral ketamine as a single dose 20–30 min prior to induction of anesthesia. Participants were young (mean age 4.4 years), and dosing ranged from 3 to 8 mg/kg (mean 5.25 mg/kg). All studies used liquid formulations diluted in cola‐flavored soft drink^23^, ^25^ or tart cherry juice^22^, ^24^ to mask the taste and improve adherence.

Across these trials, adverse effects were mostly neurological and consistent with ketamine's known pharmacodynamics. Nystagmus was the most common, reported in approximately 30% of treated children,^22^, ^23^, ^24^, ^25^ and two studies documented fasciculations in about 13% of participants.^23^, ^25^ Although these effects reached statistical significance compared with placebo, they were transient and did not interfere with the anesthesia process. No meaningful differences were found in postoperative nausea or vomiting, and no serious adverse events were reported (Table 3).

### Safety and tolerability in pain management

Regarding pain management, six studies investigated oral ketamine, including a total of 349 patients spanning both adult^18^, ^27^ and pediatric populations.^26^, ^28^, ^29^, ^30^ Two studies used single‐dose administration for procedural purposes, including neonatal circumcision^30^ and lumbar puncture‐related pain,^28^ while two used it for sedation and analgesia for laceration repair^26^, ^29^ and two used repeated dosing for postoperative^27^ or chronic pain syndromes.^18^ The age range was wide: adult and older adult samples had a mean age of 60.8 years, whereas pediatric samples varied from 14.25 days to 9.3 years. The dosing varied substantially between trials, ranging from 0.8 mg/kg/day to as high as 10 mg/kg/day. Formulations again differed, with two studies using capsules^18^, ^27^ and the remaining using liquid solutions.

Only one study used validated adverse‐event measurement tools,^27^ whereas others relied on structured questionnaires or unstructured reporting. The adverse events most frequently described – dry mouth, nausea, headache, dizziness, and sedation – were mild in intensity and generally similar in frequency to placebo. The study administering the highest dose (10 mg/kg/day) reported nystagmus in three participants (20%) and involuntary limb movements in two participants (6.7%),^23^ effects that are physiologically plausible at higher serum concentrations but reversible and not associated with clinically significant complications. No trial identified any major safety concerns, although the overall evidence base remains small (Table 4).

**Table 4:** Adverse events of oral ketamine in randomized controlled trials

Study	Population/Condition	Frequent adverse events	Treatment‐related serious AE?	Validated AE scale?
Arabzadeh *et al*., 2018^20^	Major depression	Dry mouth, somnolence, visual disturbances	No	No
Domany *et al*., 2019^21^	Major depression	Somnolence, nausea, dizziness	No (one SAE unrelated)	No
Colla *et al*., 2024^15^	Depression (ER)	Dizziness, nausea, headache	No	Yes
Glue *et al*., 2024^16^	Depression (ER)	Headache, mood worsening, noncardiac chest pain	No (urinary stone unrelated)	Yes
Smith‐Apeldoorn *et al*., 2024^11^	Depression (Esketamine)	Dizziness, anxiety, rash, dry mouth	No (one SAE unrelated)	Yes
Barkan *et al*., 2014^29^	Pediatric sedation	Sedation, dry mouth, nausea	No	No
Qureshi *et al*., 1995^26^	Pediatric procedural analgesia	Nystagmus, involuntary movements	No	No
Gutstein *et al*., 1992^23^	Pediatric premedication	Nystagmus, fasciculation, sedation	No	No
Kararmaz *et al*., 2004^24^	Pediatric premedication	Nystagmus, sedation, salivation	No	No
Şekerci *et al*., 1996^25^	Pediatric premedication	Nystagmus, fasciculation	No	No
Turhanoğlu *et al*., 2003^22^	Pediatric premedication	Nystagmus, sedation	No	No
Rayala *et al*., 2019^28^	Pediatric – LP analgesia	Sedation, somnolence	No	No
Katiyar *et al*., 2022^30^	Neonatal Circumcision	Mild sedation, reduced crying	No	No
Rabben *et al*., 1999^18^	Chronic pain	Dry mouth, dizziness	No	No
Dinsmore *et al*., 2025^27^	Postoperative pain	Nausea, dizziness, somnolence	No	Yes
Mikkelsen *et al*., 2000^17^	Healthy volunteers	Transient sedation (160 min), dizziness	No	No
Glue *et al*., 2020^31^	Healthy volunteers (ER)	Mild dissociation, dizziness	No	Yes
Qureshi *et al*., 2025^26^	Healthy volunteers	Dose‐dependent dissociation, dizziness, nausea	No	Not reported

No treatment‐related SAE was identified in any included randomized trial.

ER, extended‐release; SAE, serious adverse event.

### Safety and tolerability in healthy volunteers

Three controlled trials were conducted in healthy volunteers. Glue *et al*. evaluated an extended‐release formulation in 24 participants receiving escalating doses (60–240 mg/day) administered first as a single dose and then as repeated doses every 12 h.^31^ Mild dissociative symptoms occurred only at the highest dose. Mikkelsen *et al*.^17^ assessed 25 participants in a thermal pain model using doses of 0.5–1 mg/kg/day. Increased sedation was noted but resolved fully within approximately 160 min.^17^ Neither study reported serious adverse events, supporting the tolerability of oral ketamine in healthy individuals under controlled conditions. The most recent study, which employed a partially crossover methodology, reported 26 dissociative events, 8 episodes of dizziness, and 7 episodes of nausea among individuals receiving ketamine. Dissociative symptoms were dose‐dependent and occurred predominantly at doses exceeding 120 mg^19^ (Table 4).

### Safety and tolerability across studies

Across placebo‐controlled randomized trials, oral ketamine/esketamine was associated with higher rates of nausea (8.4% *vs*. 4.0%; RR, 2.11 [95% CI, 1.04–4.28]), dizziness (13.3% *vs*. 6.0%; RR, 2.21 [95% CI, 1.40–3.49]), sedation (39.2% *vs*. 6.4%; RR, 6.15 [95% CI, 3.58–10.56]), headache (15.4% *vs*. 9.7%; RR, 1.59 [95% CI, 1.04–2.43]), and dissociative symptoms (15.3% *vs*. 4.3%; RR, 3.52 [95% CI, 1.56–7.96]). Serious adverse events were rare, and no consistent treatment‐related safety signal was identified (Table 5).

**Table 5:** Comparative adverse events in randomized placebo‐controlled trials of oral ketamine/esketamine

Adverse Event	Ketamine/Esketamine, No./Total No. (%)	Control, No./Total No. (%)	Risk Ratio (95% CI)*	Interpretation
Nausea	32/383 (8.4)	9/227 (4.0)	2.11 (1.04–4.28)	Increased; mild and transient
Vomiting	32/296 (10.8)	23/267 (8.6)	1.26 (0.76–2.07)	Similar frequency
Dizziness (including orthostatic)	71/534 (13.3)	21/353 (6.0)	2.21 (1.40–3.49)	Consistently increased across studies
Sedation	80/204 (39.2)	13/204 (6.4)	6.15 (3.58–10.56)	Markedly increased; expected pharmacological effect
Somnolence	19/158 (12.0)	15/189 (7.9)	1.52 (0.80–2.87)	Slight increase
Headache	63/409 (15.4)	26/267 (9.7)	1.59 (1.04–2.43)	Common but nonspecific
Dissociative symptoms	47/308 (15.3)	6/138 (4.3)	3.52 (1.56–7.96)	Increased; dose‐related
Blurred vision	8/128 (6.3)	8/140 (5.7)	1.10 (0.42–2.88)	No clear difference
Anxiety/restlessness	21/212 (9.9)	3/77 (3.9)	2.54 (0.79–8.18)	Possible increase; limited precision
Fatigue	12/177 (6.8)	4/77 (5.2)	1.30 (0.44–3.82)	Similar frequency
Tremor	5/58 (8.6)	3/40 (7.5)	1.15 (0.29–4.54)	Similar frequency
Blood pressure increase	6/15 (40.0)	2/11 (18.2)	2.20 (0.55–8.73)	Limited data; possible transient increase
Dyspnea/respiratory symptoms	6/46 (13.0)	1/40 (2.5)	5.20 (0.66–40.7)	Rare but possibly increased
Elevated liver enzymes	2/17 (11.8)	0/10 (0)	NE^†^	Rare isolated finding
Serious adverse events	Rare	Rare	NE	No consistent treatment‐related signal identified

* *Risk* ratios were calculated from pooled descriptive totals across studies and should be interpreted cautiously due to heterogeneity in study populations, dosing regimens, treatment duration, and adverse‐event ascertainment methods. Estimates are based on aggregated study‐level data and should be interpreted cautiously due to heterogeneity. These estimates are exploratory and do not replace a formal meta‐analysis.

^†^ Not estimable due to zero events in the control group. Data include both spontaneously reported adverse events and systematically assessed outcomes using structured instruments, where available.

Of the 18 randomized trials included in this review, six found no statistically significant difference in the incidence of adverse events between individuals receiving oral ketamine and those receiving placebo.^15^, ^18^, ^20^, ^21^, ^27^, ^28^ Among these, two studies employed a single‐dose design,^29^, ^30^ whereas the other four administered multiple doses, ranging from 3 days to 6 weeks of treatment. However, only one of these six trials used standardized instruments specifically designed to assess adverse events, and its sample size was small (*n* = 27).^15^ The remaining studies did not apply validated scales, particularly for detecting psychiatric symptoms potentially associated with ketamine exposure.

Among the studies that reported differences between the ketamine and placebo groups, seven employed a single‐dose design,^17^, ^22^, ^23^, ^24^, ^25^, ^26^, ^28^ six of which were conducted in children. Trials using anesthetic/sedative doses (3–10 mg/kg) reported nystagmus in 13–40% of participants, involuntary limb movements in 7–13.3%, and tongue fasciculation in 20% at the 6 mg/kg dose.^22^, ^23^, ^24^, ^25^, ^26^ The remaining two studies used subanesthetic doses (0.5–1 mg/kg): one in adults reported only increased sedation in the ketamine group,^17^ while the pediatric study observed dizziness in 7.6% and nausea in 3.8% of participants.^28^

The five studies evaluating multiple‐dose regimens administered oral ketamine in daily doses ranging from 30 to 240 mg, with treatment durations varying from once daily for 3 days to three times daily for up to 42 days.^11^, ^16^, ^18^, ^19^, ^31^ Dissociative symptoms were the most frequently reported adverse effect, occurring in 45.8–100% of participants receiving 240 mg/day^16^, ^19^ and in 15.6% of those treated with 180 mg/day.^16^ Headache was reported in 12.5% of participants in Glue *et al*.^31^ and in 26.7% in Qureshi *et al*.^19^ Dizziness ranged from 8.3% to 33.3% across studies,^11^, ^19^, ^31^ with orthostatic dizziness specifically noted in 13% of participants in Smith‐Apeldoorn *et al*.^11^

## Discussion

This systematic review synthesizes evidence from 18 randomized controlled trials evaluating the safety and tolerability of oral ketamine across diverse clinical contexts including depression, pre‐anesthetic premedication, pain management, and experimental research in healthy volunteers. Although oral ketamine remains an off‐label treatment without a standardized pharmaceutical formulation, findings across controlled trials consistently suggest a favorable short‐term safety profile, with most adverse effects being mild, transient, and consistent with its established pharmacodynamic profile. These findings add to the growing literature on the clinical relevance of non‐parenteral ketamine administration, particularly in psychiatric and chronic pain disorders.

Taken together, these findings are consistent with previous systematic reviews focusing specifically on the antidepressant use of oral ketamine. Rosenblat *et al*. synthesized early clinical evidence and reported good overall tolerability of oral ketamine across heterogeneous study designs, with no clinically significant safety signals identified, although the quality of evidence was limited and adverse‐event monitoring was inconsistent.^8^ More recently, an updated systematic review by Meshkat *et al*.,^9^ including 22 clinical studies and over 2300 patients, similarly concluded that oral ketamine is generally well tolerated, with predominantly mild and transient adverse effects and a low incidence of serious adverse events, even in repeated‐dose regimens. Importantly, both reviews highlighted the absence of robust long‐term safety data and emphasized methodological limitations, including small sample sizes, heterogeneous formulations, and variable adverse‐event reporting – limitations that are also evident in the randomized controlled trials synthesized in the present review.^9^

### Comparative tolerability with other ketamine formulations

In line with prior reviews of oral ketamine for depression, the lower rates of dissociation and cardiovascular effects observed with oral administration may partly explain its favorable tolerability profile. Both Rosenblat *et al*. and Meshkat *et al*.^8^, ^9^ noted that dissociative symptoms were uncommon or delayed in oral ketamine trials and typically emerged only after repeated dosing or at higher total daily doses, contrasting with the rapid and prominent psychotomimetic effects reported with intravenous or intranasal formulations.

Recent reviews comparing ketamine delivery routes indicate that oral administration exhibits a lower incidence of acute dissociation, hypertension, and psychotomimetic effects when compared with intravenous (IV) and intranasal forms.^32^, ^33^ This difference is attributed to the slower absorption rate and extensive first‐pass metabolism leading to higher norketamine‐to‐ketamine plasma ratios, potentially conferring *reduced psychotomimetic risk* but also *more variable therapeutic potency*.^4^

In contrast to intranasal esketamine, where dissociative symptoms are reported in up to 75% of patients and acute blood pressure elevations are common,^34^ oral ketamine trials in depression reviewed here reported dissociation as absent or minimal, appearing primarily at high doses (>120–180 mg/day) in healthy‐volunteer studies. Cardiovascular effects were rare, mild, and nonsignificant between treatment and placebo arms – consistent with the greater hemodynamic stability observed in oral and sublingual formulations.^7^, ^35^

### Adverse events and organ‐specific safety considerations

Across all included RCTs, no treatment‐related serious adverse events were reported, and withdrawal due to adverse effects was rare. The most frequently observed reactions were predictable, including nausea, dizziness, dry mouth, blurred vision, and transient sedation. Liver toxicity, frequently raised as a concern in chronic ketamine misuse, was limited to benign transient enzyme elevations without clinical hepatitis, aligning with findings from longer non‐RCT cohorts in pain and psychiatric use.^36^, ^37^

Urological toxicity, a major risk in recreational ketamine exposure, was rare; a single case of urinary calculi occurred in a placebo‐controlled extended‐release study and was deemed unrelated. Large‐scale ketamine misuse cohorts consistently indicate that uropathy risk is dose‐dependent and associated with chronic exposure to high cumulative doses – not commonly reached in therapeutic oral administration. Taken together, this suggests that oral therapeutic regimens, particularly at antidepressant ranges (30–120 mg/day), may confer a substantially lower urological risk, although long‐term controlled studies remain a priority for the field.

### Pediatric and procedural applications

In procedural sedation and pediatric premedication, oral ketamine showed good tolerability, with no serious adverse events reported among 239 children; adverse effects were limited to nystagmus, transient neuromuscular fasciculations, and mild sedation. Comparatively, IV ketamine in pediatric sedation has a higher frequency of recovery agitation and vomiting,^38^ suggesting that the oral route may offer a more stable recovery profile at the cost of slower onset and greater interindividual pharmacokinetics.^39^

### Methodological limitations and future directions

Our strict inclusion of RCTs focused the evidence on short‐term safety and tolerability under controlled exposure conditions. However, this methodological choice represents a trade‐off in external validity. RCTs are often underpowered to identify rare, cumulative, or long‐term complications. Consequently, risks such as chronic urological toxicity, persistent hepatobiliary changes, or potential for dependence – which often emerge after prolonged real‐world use – may not be fully represented in this synthesis. Future safety assessments should integrate data from large‐scale pharmacovigilance studies and long‐term observational cohorts to complement the controlled randomized evidence summarized here.

These limitations mirror those identified in previous systematic reviews of oral ketamine for depression,^8^, ^9^ which consistently underscored the need for standardized oral formulations, systematic adverse‐event assessment, and longer follow‐up periods to adequately characterize cumulative risks. Together, the convergence of findings from depression‐focused reviews and the broader safety‐oriented randomized evidence presented here reinforces the conclusion that oral ketamine appears acutely safe and well tolerated, while simultaneously underscoring the critical gaps that must be addressed before widespread clinical adoption.

Cardiovascular outcomes showed slightly higher certainty because several studies used objective physiological measurements such as blood pressure and heart rate monitoring. However, the available evidence remains largely restricted to short‐term exposure. Therefore, the long‐term safety profile of oral ketamine cannot be established based on the currently available randomized trials and warrants further investigation in larger and longer‐duration studies.

The included studies showed considerable clinical heterogeneity across several dimensions, including study populations, clinical indications, dosing regimens, and duration of exposure. These differences limited the feasibility of performing quantitative heterogeneity assessments such as *I* ^2^ statistics and further supported the use of a narrative synthesis approach.

Despite the overall consistency of safety findings, the current evidence base is limited by small sample sizes, heterogeneous oral formulations, and relatively short follow‐up periods. Few RCTs extended beyond 6–12 weeks, leaving uncertainty about long‐term safety, particularly regarding cumulative hepatotoxicity, urological risk, and chronic neuropsychiatric outcomes. These findings support the short‐term safety of oral ketamine under controlled conditions but should not be interpreted as evidence of long‐term safety in real‐world use.

An important methodological limitation of the included trials relates to heterogeneity in adverse‐event assessment. Several studies relied on unstructured reporting or spontaneous participant complaints rather than systematic monitoring using validated instruments. The inclusion of structured assessments in some trials suggests that symptoms such as dizziness, orthostatic intolerance, and dissociative effects may be detected more frequently when systematic instruments are used. Consequently, although the available randomized evidence suggests a favorable short‐term safety profile, some adverse effects – particularly subtle cognitive or neuropsychiatric symptoms – may have been underreported. This limitation should be considered when interpreting the apparent tolerability of oral ketamine.

Future research should prioritize:

- Standardization of oral ketamine formulations, including development of commercially available preparations and extended‐release versions,
- Longitudinal studies with extended duration to monitor long‐term adverse effects, especially hepatobiliary and urological outcomes,
- Systematic use of validated adverse‐event measurement tools, particularly for dissociative, cognitive, and neuropsychiatric symptoms, and
- Dose–response modeling that incorporates norketamine metabolism to clarify efficacy–tolerability relationships.

## Conclusions

In conclusion, oral ketamine demonstrates a favorable short‐term safety and tolerability profile in randomized controlled settings, with predominantly mild and transient adverse events and no treatment‐associated serious harm across psychiatric, analgesic, pediatric, and experimental contexts. However, the current evidence base is insufficient to establish long‐term safety, particularly regarding cumulative hepatobiliary, urological, and neuropsychiatric risks. These findings should therefore be interpreted as evidence of acute and subacute tolerability rather than definitive long‐term safety. Standardized formulations, systematic adverse‐event monitoring, and longer‐duration studies are needed before broader clinical adoption.

## Author contributions

N.N. and M.H.N.C. conceived and designed the study. N.N. and R.P. performed literature screening and data extraction. J.E.H., R.G.S., and M.H.N.C. critically revised the manuscript for important intellectual content. All authors approved the final manuscript.

## Funding statement

This study was financed in part by the Brazilian fostering agency Coordination for the Advancement of Higher Education Personnel (Coordenação de Aperfeiçoamento de Pessoal de Nível Superior – CAPES), finance code 001. M.H.N.C. is the recipient of a CNPq Research Productivity Fellowship (process no. 309870/2025‐9). N.N. is a recipient of a CAPES doctoral scholarship.

## Disclosure statement

The authors have no conflicts of interest declare.

## Data Availability

Data sharing not applicable to this article as no datasets were generated or analysed during the current study.

## Appendix I Detailed Search Strategy

A systematic search of the literature was conducted in the following electronic databases:

- PubMed
- Embase
- Scopus
- Web of Science

The search strategy combined terms related to ketamine, oral administration, and safety outcomes using Boolean operators. The strategy included synonyms and variations of search terms to maximize sensitivity and was adapted for each database as appropriate. No restrictions on publication year were applied. In addition, the reference lists of included studies and relevant review articles were manually screened to identify additional eligible studies.

### Search strategy

(‘Ketamine’ OR ‘Ketamine hydrochloride’ OR ‘Racemic ketamine’ OR ‘Esketamine’ OR ‘S‐ketamine’ OR ‘(S)‐ketamine’)

AND

(‘Oral administration’ OR ‘Oral route’ OR ‘Tablet’)

AND

(‘Safety’ OR ‘Tolerability’ OR ‘Adverse effects’ OR ‘Side effects’ OR ‘Adverse events’ OR ‘Treatment‐emergent adverse events2019x OR ‘Acceptability’)
